# Risk factors for 5-year complications after midurethral sling surgery for stress urinary incontinence: a retrospective cohort study from Taiwan

**DOI:** 10.1038/s41598-023-48558-8

**Published:** 2023-12-05

**Authors:** Tai-Fu Chou, Ying-Fang Hsia, Tseh-Lee Hwang, Wu-Chou Lin, Daniel Tzu-Li Chen, Chien-Fong Huang, Chin-Chi Kuo, Huey-Yi Chen, Hsiu-Yin Chiang

**Affiliations:** 1 Department of Obstetrics and Gynecology, China Medical University Hospital, Taichung, 40447 Taiwan; 2 Big Data Center, China Medical University Hospital, Taichung, 40447 Taiwan; 3 Kidney Institute, China Medical University Hospital, Taichung, Taiwan; 4 Department of Medical Research, China Medical University Hospital, Taichung, Taiwan; 5 School of Chinese Medicine, College of Chinese Medicine, China Medical University, Taichung, Taiwan; 6 Graduate Institute of Biomedicine, College of Medicine, China Medical University, Taichung, Taiwan; 7 Department of Psychiatry and Mind-Body Interface Laboratory, China Medical University Hospital, Taichung, Taiwan

**Keywords:** Endocrinology, Medical research, Risk factors, Urology

## Abstract

Midurethral sling surgery is the current gold standard worldwide for stress urinary incontinence (SUI) surgery, with over 90% of surgeons worldwide using the midurethral sling for SUI between 2008 and 2018. However, concerns surround mesh-related adverse events associated with the midurethral sling. The decision to use the midurethral sling for surgical treatment has become a challenging one for clinicians, surgeons and patients. We sought to determine the factors for 5-year complications after midurethral sling surgery, to improve the clinical decision-making process. Records were reviewed from a total of 1961 female patients who underwent their first midurethral sling surgery for SUI between 2003 and 2018 at a single teaching hospital in Taiwan. A multivariable Cox proportional hazard model calculated the hazard ratios of risk factors for surgical complications, after adjusting for confounders. Surgical complications (i.e., secondary surgery and urinary retention) occurred in 93 (4.7%) patients within 5 years following the index operations. These patients were more likely to be older, to have a history of menopausal syndrome within 1 year prior to the index operation, a medication history of oral antidiabetic drug use, hormone replacement therapy (HRT), slower average flow rate, and longer voiding time compared with patients without surgical complications. In the multivariate analysis, HRT (adjusted hazard ratio, 1.787; 95% confidence interval, 1.011–3.158, p = 0.04) was significantly associated with surgical complications at 5 years, after adjusting for age, gender, diabetes, menopause syndrome, average flow rate, and sling type. Our findings suggest that a medication history of HRT may be a risk factor associated with surgical complications, especially urinary retention, at 5 years in women undergoing midurethral sling surgery for SUI.

## Introduction

Stress urinary incontinence (SUI) is defined as the involuntary loss of urine during physical activity, such as coughing, sneezing, laughing, or exercising, that may increase abdominal pressure, and SUI has been a critical public health issue^1^. SUI impacts enormously on the patient’s lifestyle and quality of life, at considerable financial cost for both patients and the health care system^2–6^. According to the European Menopause and Andropause Society (EMAS) clinical guide, the increasing prevalence of urinary incontinence and other lower urinary tract symptoms (LUTS) after menopause has been observed, and these clinical inconveniences affect 38% to 55% of women aged over 60 years^7^. International guidance recommends conservative therapies as the first-line management of women with SUI, such as lifestyle changes and behavioral therapies, weight loss, and supervised pelvic floor muscle training^8,9^. For patients who do not experience improvement in physical symptoms and quality of life with nonsurgical management or pharmacotherapy, surgery is the next step^8,9^. The current gold standard for SUI surgery is the midurethral sling, which has been supported by the American Urogynecologic Society (AUGS) and the Society of Urodynamics, Female Pelvic Medicine and Urogenital Reconstruction (SUFU)^10^, and Royal Australian and New Zealand College of Obstetricians and Gynaecologists (RANZCOG)^11^. Between 2008 and 2018, over 90% of surgeons worldwide using the midurethral sling for SUI^12,13^.

Nonetheless, mesh-related adverse events are associated with the midurethral sling^14,15^. In response to surgical complications involving the use of transvaginal mesh for pelvic organ prolapse, the United States Food and Drug Administration (US FDA) issued a safety communication in 2011^16^ and, since April 16, 2019, has banned the production and sale of transvaginal mesh for pelvic organ prolapse^17^. By early 2015, over 70,000 women in the US had filed lawsuits alleging complications associated with transvaginal mesh used for both SUI and pelvic organ prolapse surgical procedures^18^. Litigation suits relating to financial costs and safety concerns surrounding these procedures have increased general awareness amongst the public and medical community of all synthetic mesh use in pelvic floor disorders, including SUI^18^. However, it is important to note that the US FDA publications did not refer to traditional midurethral slings as the subject of their safety communication; their 2019 advice stated that full-length midurethral slings are supported by 1-year follow-up safety and efficacy data from clinical trials and that longer-term follow-up data are also available, but are from a smaller pool of evidence^8^. Nevertheless, the decision to use the midurethral sling for surgical treatment has become a challenging one for clinicians, surgeons and patients. With this in mind, we sought to determine the risk factors, especially age, menopausal syndrome and hormone replacement therapy (HRT), for 5-year complications after midurethral sling surgery, to improve the clinical decision-making process.

## Materials and methods

### Study population

The China Medical University Hospital–Clinical Research Data Repository (CMUH–CRDR) was established by the Big Data Center of CMUH, which contains the medical records of 2,873,887 patients who sought care in CMUH between 2003 and 2018. The CMUH–CRDR has been described in greater detail in previous articles^19,20^. All patients enrolled in the CMUH–CRDR were followed up until December 31, 2018, or death, whichever occurred earlier. This study was approved with waived informed consent by the Big Data Center of CMUH and the Research Ethical Committee/Institutional Review Board of CMUH (CMUH105-REC3-068 & CMUH111-REC3-138).

**Figure 1:**
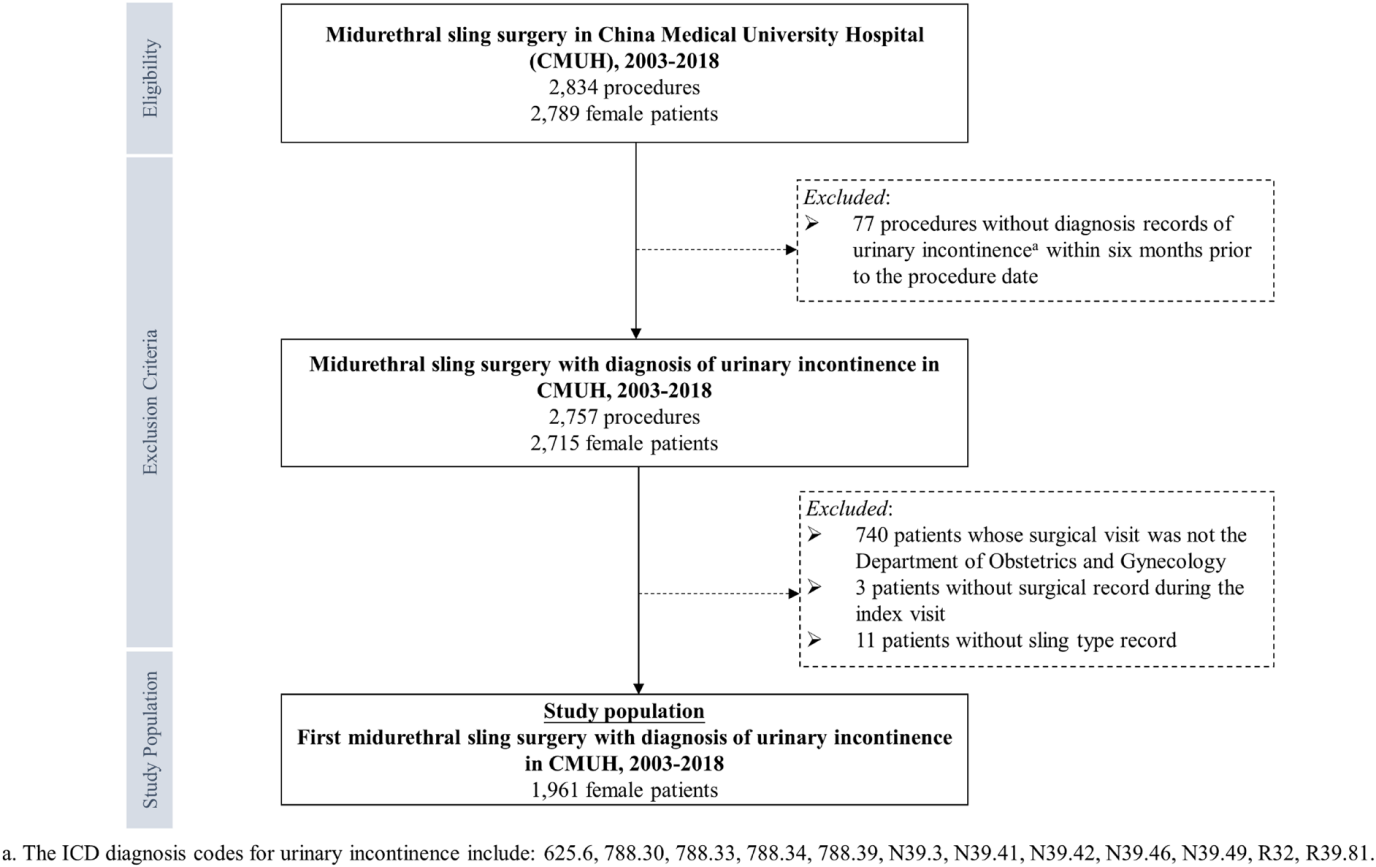
The selection process of the study population.

In this retrospective cohort study, we identified 2789 patients from the CMUH–CRDR records who underwent 2834 midurethral sling surgery procedures between 2003 and 2018. Miduretheral sling surgeries included in this study were tension free vaginal tape (TVT) or trans-obturator tension free vaginal tape (TVTO), which are the current gold standard surgical treatment^8,9^. Next, we excluded operations without SUI diagnosis records during the index visit or within six months prior to the procedure date, patients whose surgical visits were not in the Department of Obstetrics and Gynecology, and patients who did not have any surgical records or who did not have sling type records on their surgical notes during the index visit (Fig. 1; Supplementary Table 1). The final study population consisted of 1961 female patients with a SUI diagnosis who underwent their first midurethral sling surgery (index procedure) between 2003 and 2018 in the Department of Obstetrics and Gynecology of CMUH.

### Definition of risk factors

Demographic information, comorbidities, medication histories, biochemical profiles, reports of urodynamic testing and surgical procedures were collected from the CMUH–CRDR. Comorbidities of diabetes mellitus, hypertension, cardiovascular diseases, psychosis, menopausal syndrome and constipation were defined by the International Classification of Diseases, 9th/10th Revisions, Clinical Modification (ICD-9-CM and ICD-10-CM) codes, and medication histories for hypertension, diabetes, and hormone replacement therapy (HRT) were recorded within 1 year before the index midurethral sling surgery (Supplementary Table 1). Biochemical profiles of blood glucose, liver function, renal function, and complete blood count were obtained within 1 year prior to the index procedures. Reports of urodynamic testing, such as uroflowmetry information and urethra pressure profiles, were obtained within 3 years prior to the index procedures. Surgical information extracted from the operation database included length of operation, American Society of Anesthesiology (ASA) score, wound contamination classification, anesthesia type, and sling type.

### Definition of outcomes

The primary outcome of interest was surgical composite complications (including secondary surgery and urinary retention) within 5 years following the index procedures. Secondary surgery was defined as the patient undergoing another operation (i.e., TVT/TVTO) within 5 years due to recurrent SUI; urinary retention was defined by ICD-9-CM and ICD-10-CM codes (Supplementary Table 1).

### Statistical analysis

Continuous variables are presented as medians and interquartile ranges (IQRs); categorical variables are reported as frequencies and proportions (%). The associations between surgical complications and covariates were analyzed using the Wilcoxon rank-sum test (nonparametric) for continuous variables and the chi-square test for categorical variables.

We evaluated the associations of the risk factors with the risk of 5-year surgical complications by using a competing risk analysis with deaths considered censoring events using cause-specific Cox proportional hazards modeling and used age as the time scale. We included age, diabetes mellitus, duration of surgery, menopausal syndrome, HRT, average flow rate (AFR), voiding time and sling type in the Cox model. Subgroup analyses were performed for patients categorized by age groups and diabetes status. The Kaplan–Meier method was employed to generate time-to-event curves throughout the study period, and the log-rank test was used to assess differences between the curves of different subgroups.

All statistical analyses were performed using SAS version 9.4 (SAS Institute Inc., Cary, NC, USA). The 2-sided statistical significance level was α = 0.05.

## Results

We identified 1961 women with SUI who underwent first-time midurethral sling surgery between 2003 and 2018 in CMUH, with a median age of 53.5 years (IQR, 46.4–64.0; Table 1). Of them, 21.8% were concomitant procedure with pelvic floor reconstruction and 16.7% with hysterectomy. Surgical complications (i.e., secondary surgery and urinary retention) occurred in 93 (4.7%) patients within 5 years following the index operations. The mean follow-up time after the miduretheral sling surgery was 1754 days (standard deviation, 340 days).

**Table 1:** Demographic and clinical characteristics of patients with stress urinary incontinence who received surgical treatment.

Characteristics^a^	Available N (%)	Overall (N = 1961)	Patients without 5-year surgical complications (N = 1868)	Patients with 5-year surgical complications^b^ (N = 93)	*P*-value^c^
Demographic information					
Age (years)	1961 (100.0)	53.5 (46.4, 64.0)	53.1 (46.3, 63.7)	60.3 (51.4, 68.8)	< 0.0001
Age > 65 years		438 (22.3)	400 (21.4)	38 (40.9)	< 0.0001
BMI (kg/m^2^)	844 (43.0)	24.5 (22.3, 27.1)	24.5 (22.3, 27.1)	25.0 (22.8, 28.0)	0.3993
Baseline comorbidities^d^	1961 (100.0)				
Diabetes mellitus		178 (9.1)	165 (8.8)	13 (14.0)	0.0918
Hypertension		274 (14.0)	259 (13.9)	15 (16.1)	0.5388
Cardiovascular diseases		102 (5.2)	95 (5.1)	7 (7.5)	0.3008
Psychosis					
Depression		35 (1.8)	32 (1.7)	3 (3.2)	0.2822
Anxiety		82 (4.2)	80 (4.3)	2 (2.2)	0.3161
Schizophrenia		1 (0.1)	1 (0.1)	0 (0.0)	0.8234
Affective psychosis		33 (1.7)	30 (1.6)	3 (3.2)	0.2359
Organic psychosis		18 (0.9)	16 (0.9)	2 (2.2)	0.2016
Other psychosis		54 (2.8)	51 (2.7)	3 (3.2)	0.7756
Menopausal syndrome		194 (9.9)	179 (9.6)	15 (16.1)	0.0390
Constipation		140 (7.1)	131 (7.0)	9 (9.7)	0.3300
History of medication use^e^	1961 (100.0)				
Antihypertensive					
ACEI		42 (2.1)	42 (2.3)	0 (0.0)	0.1438
ARB		104 (5.3)	99 (5.3)	5 (5.4)	0.9743
Diuretic		660 (33.7)	632 (33.8)	28 (30.1)	0.4581
Antidiabetic					
OAD		80 (4.1)	72 (3.9)	8 (8.6)	0.0239
Insulin		164 (8.4)	153 (8.2)	11 (11.8)	0.2162
Hormone replacement therapy		285 (14.5)	255 (13.7)	30 (32.3)	< 0.0001
Baseline biochemical profiles^f^					
Glucose AC (mg/dL)	1883 (96.0)	97.0 (88.0, 114.0)	97.0 (88.0, 114.0)	100.0 (89.0, 112.0)	0.9365
Glucose random (mg/dL)	420 (21.4)	122.0 (105.0, 154.0)	121.0 (105.0, 154.0)	134.0 (108.0, 157.0)	0.2876
AST (IU/L)	1951 (99.5)	22.0 (19.0, 26.0)	22.0 (19.0, 27.0)	22.0 (19.0, 25.0)	0.4061
ALT (IU/L)	1953 (99.6)	19.0 (15.0, 26.0)	19.0 (15.0, 26.0)	20.0 (15.0, 23.0)	0.6926
CBC-I					
WBC (10^3^/μL)	1956 (99.7)	6.5 (5.4, 7.7)	6.5 (5.4, 7.7)	6.4 (5.6, 7.5)	0.7959
RBC (10^6^/μL)	1954 (99.6)	4.4 (4.2, 4.7)	4.4 (4.2, 4.7)	4.4 (4.1, 4.7)	0.1403
Hemoglobin (g/dL)	1959 (99.9)	13.0 (12.2, 13.7)	13.0 (12.2, 13.7)	13.1 (12.1, 13.6)	0.7698
HCT (%)	1954 (99.6)	38.9 (36.6, 40.7)	38.9 (36.6, 40.7)	38.6 (36.9, 40.5)	0.5898
Platelet count (10^3^/μL)	1954 (99.6)	245.0 (207.0, 287.0)	245.0 (208.0, 288.0)	234.0 (195.0, 281.0)	0.1011
MCV (fL)	1953 (99.6)	88.3 (84.0, 91.3)	88.3 (83.9, 91.2)	88.9 (86.1, 91.5)	0.0920
MCH (pg)	1953 (99.6)	29.7 (28.0, 30.8)	29.7 (28.0, 30.8)	30.0 (28.9, 30.8)	0.1665
MCHC (g/dL)	1953 (99.6)	33.5 (32.7, 34.1)	33.5 (32.7, 34.1)	33.6 (32.8, 34.2)	0.6849
Serum creatinine (mg/dL)	1955 (99.7)	0.7 (0.6, 0.8)	0.7 (0.6, 0.8)	0.7 (0.6, 0.8)	0.0609
eGFR (mL/min/1.73m^2^)^g^	1955 (99.7)	99.2 (85.6, 107.3)	99.5 (86.3, 107.5)	91.8 (76.7, 101.8)	< 0.0001
CKD Stage	1955 (99.7)				0.0428
1, 2 (eGFR ≥ 60)		1869 (95.6)	1784 (95.8)	85 (91.4)	
3, 4, 5 (eGFR < 60)		86 (4.4)	78 (4.2)	8 (8.6)	
Surgical information					
Surgical duration (h)	1961 (100.0)	1.5 (0.8, 3.0)	1.4 (0.8, 3.0)	2.2 (1.0, 3.0)	0.0831
ASA score ≥ 3	1781 (90.8)	148 (8.3)	140 (8.2)	8 (10.0)	0.5752
Wound contamination class	1736 (88.5)				0.7629
Clean		139 (8.0)	134 (8.1)	5 (6.4)	
Clean contaminated		1462 (84.2)	1394 (84.1)	68 (87.2)	
Contaminated		135 (7.8)	130 (7.8)	5 (6.4)	
General anesthesia		1937 (98.8)	1848 (98.9)	89 (95.7)	0.0057
Sling type	1758 (89.6)				0.0777
TOT		620 (35.3)	601 (35.7)	19 (25.7)	
TVT-O		1138 (64.7)	1083 (64.3)	55 (74.3)	
Report of urodynamic testing^h^					
Uroflowmetry					
Maximum flow rate (ml/s)	1846 (94.1)	21.0 (16.4, 26.1)	21.0 (16.6, 26.2)	19.1 (14.0, 26.0)	0.0846
Average flow rate (ml/s)	1846 (94.1)	10.0 (7.0, 12.3)	10.0 (7.0, 12.4)	8.0 (5.0, 12.0)	0.0004
Voiding volume (ml)	1846 (94.1)	256.5 (211.0, 307.0)	257.0 (212.0, 307.0)	249.5 (200.0, 311.0)	0.4712
Voiding time (s)	1846 (94.1)	33.6 (23.6, 48.4)	33.0 (23.6, 47.8)	45.4 (30.4, 64.4)	< 0.0001
Residual urine or residual volume (ml)	1846 (94.1)				
Median (Q1, Q3)		10.0 (5.0, 10.0)	10.0 (5.0, 10.0)	10.0 (5.0, 30.0)	0.0001
Mean (standard deviation)		15.7 (26.4)	14.9 (24.9)	30.8 (44.8)	0.0001
Urethra pressure profile					
Maximum urethral closure pressure (cm H_2_O)	1844 (94.0)	58.0 (45.0, 76.0)	58.0 (44.0, 76.0)	58.0 (45.0, 73.0)	0.7728
Functional length (cm)	1843 (94.0)	3.3 (2.8, 3.9)	3.3 (2.8, 3.9)	3.2 (2.8, 3.8)	0.7651
Length of continence zone (cm)	1665 (84.9)	1.8 (1.3, 2.1)	1.8 (1.3, 2.1)	1.7 (1.4, 2.1)	0.9823

^a^Categorical variables are presented as frequencies (%) and continuous variables are presented as medians (Q1, Q3), if not otherwise specified.

^b^The 5-year surgical complications included secondary surgery and urine retention.

^c^*P*-values are calculated by the Wilcoxon rank-sum test for continuous variables and the chi-square test for categorical variables.

^d^Diabetes mellitus and hypertension were defined as having the respective diagnosis and medication within 1 year before the index date. Cardiovascular diseases, psychosis, menopause syndrome and constipation were defined by the diagnosis within 1 year before the index date. Stroke history was defined as having the diagnosis before the index date. Detailed definitions are listed in the Supplemental Table 1.

^e^Medication use within 1 year before the index date.

^f^Biochemical values measured within 1 year prior to and closest to the index date.

^g^eGFR is calculated using the CKD-EPI Eq. (141 × min(S-Cre/κ, 1)α × max(S-Cre/κ, 1) − 1.209 × 0.993age × 1.018 [if female] × 1.159 [if black]).

^h^Urodynamic testing within the 3-year period prior to and closest to the index date.

ACEI, angiotensin-converting enzyme inhibitor; ALT, alanine aminotransferase; ARB, angiotensin receptor blocker; ASA, American society of anesthesiologists; AST, aspartate aminotransferase; BMI, body mass index; CBC, complete blood count; CKD, chronic kidney disease; Glucose AC, glucose ante cibum; h, hours; HCT, hematocrit; MCH, mean corpuscular hemoglobin; MCHC, mean corpuscular hemoglobin concentration; MCV, mean corpuscular volume; OAD, oral antidiabetic agent; RBC, red blood cell; TOT, transobturator tape; TVT-O, tension-free vaginal tape-obturator; WBC, white blood cell.

Patients with surgical complications were more likely to be older (60.3 years vs 53.1 years), to have a history of menopausal syndrome within 1 year prior to the index operation (16.1% vs 9.6%), a medication history of oral antidiabetic drug (OAD; 8.6% vs 3.9%), HRT (32.3% vs 13.7%), slower AFR (8.0 ml/s vs 10.0 ml/s), and longer voiding time (45.4 s vs 33.0 s), compared with patients without 5-year surgical complications.

**Figure 2:**
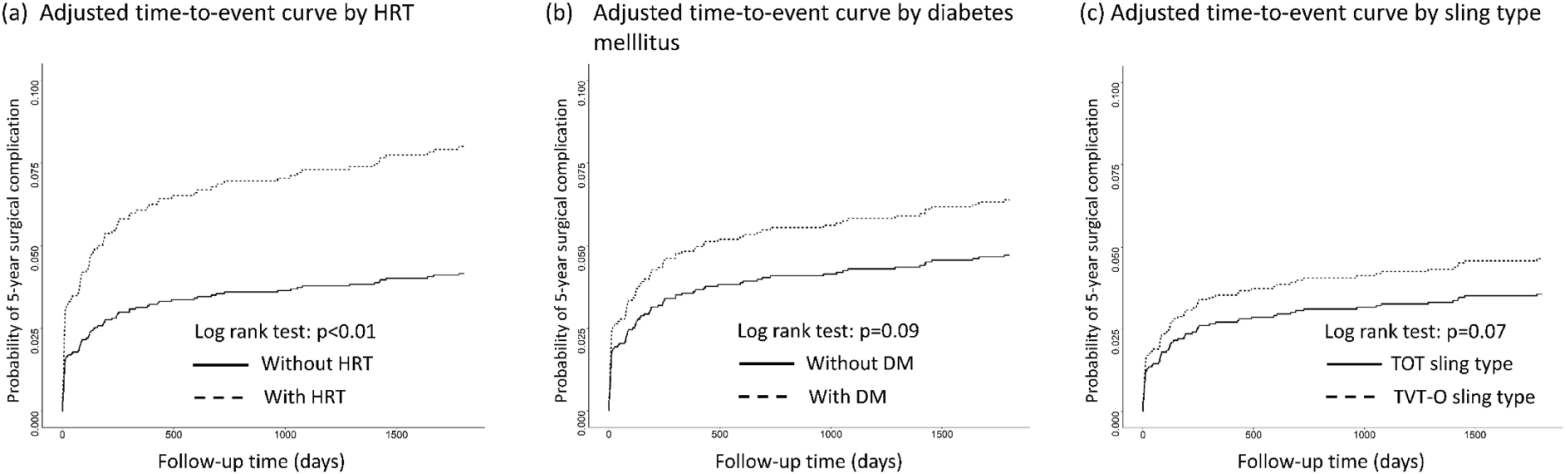
The adjusted time-to-event curve of 5-year surgical complications following the midurethral sling surgery, stratified by hormone replacement therapy, diabetes, and sling type.

We used multivariable Cox proportional hazard modeling to investigate the risk factors associated with 5-year surgical complications (Table 2). After including age, diabetes mellitus, duration of surgery, menopausal syndrome, HRT, AFR, and sling type in the full model, only HRT (adjusted HR, 1.787; 95% CI, 1.011–3.158, p = 0.04) remained significantly associated with the risk of 5-year surgical complications. Similarly, when combining menopausal syndrome and HRT as one variable in the model, the effect size stayed significant (aHR, 1.794; 95% CI, 1.033–3.114; Supplementary Table 2). The adjusted time-to-event curves demonstrated that patients with HRT had a significant higher probability of developing 5-year surgical complications than those without menopausal syndrome or HRT (Fig. 2).

**Table 2:** Hazard ratios (95% confidence intervals) of risk factors associated with 5-year surgical complications after midurethral sling surgery.

Characteristic	Available N	No. of events	HR (95% CI)	
			Crude model	Full model^a^
Age (year)	1961	93	1.034 (1.018, 1.050)*	1.016 (0.994, 1.040)
Diabetes mellitus				
No	1783	80	Ref	Ref
Yes	178	13	1.642 (0.917, 2.942)	1.516 (0.756, 3.040)
Surgical duration (h)	1961	93	1.114 (0.985, 1.261)	1.070 (0.901, 1.271)
Menopause syndrome diagnosis				
No	1767	78	Ref	Ref
Yes	194	15	1.779 (1.025, 3.088)*	1.335 (0.673, 2.650)
Hormone replacement therapy				
No	1676	63	Ref	Ref
Yes	285	30	2.929 (1.895, 4.527)*	1.787 (1.011, 3.158)*
Average flow rate (ml/s)	1846	90	0.904 (0.846, 0.965)*	0.964 (0.892, 1.042)
TVT-O sling type				
TOT	620	19	Ref	Ref
TVT-O	1138	55	1.596 (0.949, 2.686)	1.292 (0.716, 2.331)

CI, confidence interval; HR, hazard ratio; TOT, transobturator tape; TVT-O, tension-free vaginal tape-obturator.

^a^Full model: adjusted for age, diabetes mellitus, surgical duration, menopause syndrome diagnosis, hormone replacement therapy, average flow rate and TVT-O sling type (n = 1653).

*Statistically significant.

Subgroup analyses of patients characterized by age (≤ 65 years, > 65 years) and diabetes status showed that HRT remained associated with adverse outcomes in patients without diabetes (aHR, 2.239; 95% CI, 1.235–4.057; p for interaction = 0.0807) (Table 3).

**Table 3:** Adjusted hazard ratios of 5-year surgical complications after midurethral sling surgery, stratified by subgroups of age and diabetes mellitus.

	Available N	No. of events	HR (95% CI)
Age ≤ 65 years			
Diabetes mellitus			
No	1429	50	Ref
Yes	94	5	1.793 (0.669, 4.807)
Surgical duration (h)	1523	55	1.111 (0.912, 1.355)
Menopausal syndrome diagnosis			
No	1389	45	Ref
Yes	134	10	2.222 (0.982, 5.029)
Hormone replacement therapy			
No	1360	42	Ref
Yes	163	13	1.384 (0.596, 3.215)
Average flow rate (ml/s)	1423	55	0.929 (0.853, 1.012)
TVT-O sling type			
TOT	475	12	Ref
TVT-O	885	32	1.235 (0.598, 2.547)
Age > 65 years			
Diabetes mellitus			
No	354	30	Ref
Yes	84	8	1.185 (0.476, 2.952)
Surgical duration (h)	438	38	0.966 (0.697, 1.338)
Menopausal syndrome diagnosis			
No	378	33	Ref
Yes	60	5	0.618 (0.146, 2.618)
Hormone replacement therapy			
No	316	21	Ref
Yes	122	17	2.131 (0.880, 5.163)
Average flow rate (ml/s)	423	35	1.036 (0.893, 1.201)
TVT-O sling type			
TOT	145	7	Ref
TVT-O	253	23	1.392 (0.516, 3.754)
Diabetes mellitus			
Age (year)	178	13	1.013 (0.959, 1.069)
Surgical duration (h)	178	13	1.630 (0.971, 2.735)
Menopausal syndrome diagnosis			
No	162	12	Ref
Yes	16	1	0.000 (0.000, 0.000)
Hormone replacement therapy			
No	143	10	Ref
Yes	35	3	0.340 (0.036, 3.207)
Average flow rate (ml/s)	170	12	1.049 (0.923, 1.193)
TVT-O sling type			
TOT	83	4	Ref
TVT-O	87	8	1.249 (0.400, 3.895)
Non-diabetes mellitus			
Age (year)	1783	80	1.015 (0.989, 1.041)
Surgical duration (h)	1783	80	1.023 (0.847, 1.235)
Menopausal syndrome diagnosis			
No	1605	66	Ref
Yes	178	14	1.504 (0.743, 3.046)
Hormone replacement therapy			
No	1533	53	Ref
Yes	250	27	2.239 (1.235, 4.057)*^,^^a^
Average flow rate (ml/s)	1676	78	0.940 (0.860, 1.028)
TVT-O sling type			
TOT	537	15	Ref
TVT-O	1051	47	1.191 (0.611, 2.321)

CI, confidence interval; HR, hazard ratio; TOT, transobturator tape; TVT-O, tension-free vaginal tape-obturator.

*Statistically significant.

^a^*P* for interaction with hormone replacement therapy was 0.0807.

When separating the composite 5-year surgical outcomes into secondary surgery and urinary retention, the HRT (aHR, 1.904; 95% CI, 1.037–3.496) and TVT-O sling type (aHR, 2.389; 95% CI, 1.048–5.448) were significant risk factors and average flow rate (aHR, 0.899; 95% CI, 0.817–0.989) was significant protective factor of urinary retention (Supplementary Table 3).

## Discussion

This large retrospective cohort study found that 4.7% of patients receiving miduretheral sling surgery had unfavorable composite complications (i.e., urinary retention or secondary surgery) within 5 years. History of HRT was the only significant risk factor of 5-year surgical complication, with an almost two-fold increased risk. The association between HRT remained significant in the subgroup of patients without diabetes. In addition, HRT was significantly associated with increased risk of urinary retention when we did separate analysis on surgical complication. Several interesting findings are worth for discussion and further investigation.

### Surgical complications following midurethral sling surgery

A global review of epidemiological research on SUI that included studies published between January 1980 and October 2002 reported a median prevalence of female UI of 27.6%, most commonly caused by stress (50%)^21^. Population-based prevalence rates of SUI among Chinese women range from 6.7 to 44%^22^, while one Taiwanese study has reported a prevalence of 18.0% based on patients’ perceptions^23^.

Midurethral sling surgery is the current gold standard worldwide for SUI surgery; however, concerns surround mesh-related adverse events associated with the midurethral sling. One retrospective cohort study that identified 188,454 eligible women who underwent an index sling surgery showed that the 9-year risk of sling revision/removal was relatively low at 3.7%, with a 9-year risk of 1.3% (95% CI, 1.2–1.4) for urinary retention and the majority occurred within 4 years after the surgery^24^. Similarly, in our study, a relatively low proportion of patients (4.7%) developed surgical complications within 5 years of undergoing midurethral surgery.

### Risk factors associated with surgical complications

We identified factors that potentially place patients at risk of experiencing surgical complications after midurethral sling surgery and are worthy for being discussed.

#### Menopausal syndrome

A 2012 review of the literature on the epidemiology of UI in women and the effects of HRT on urinary leakage found that UI was a common symptom during menopause^25^. Decreased estrogen concentrations associated with menopause have been considered to be responsible for the increasing prevalence of SUI in aging women, possibly because vaginal tissue is weaker in postmenopausal women than in premenopausal women and thus becomes a risk factor for the deterioration of continence mechanisms and consequently the efficacy of anti-incontinence surgery^26,27^. Although menopausal syndrome is a recognized risk factor for SUI, scant study evidence describes the effects of menopausal syndrome on surgical outcomes. A retrospective study from Turkey that investigated mesh erosion after tension free vaginal tape (TVT) and transobturator tape (TOT) found that menopausal status was a statistically significant factor in patients with mesh erosion, but was no longer a significant independent risk factor after multivariate analysis^28^. In contrast, our study found that patients who had a history of menopausal syndrome within 1 year prior to the index operation were more likely to develop surgical complications. Moreover, menopausal syndrome or HRT were significantly associated with the risk of 5-year surgical complications (aHR, 1.794; p = 0.0390), although the association was not significant in patients aged ≤ 65 years (aHR, 2.164; p for interaction = 0.3750). Estrogen increases angiogenesis, which is important for nourishing vaginal tissue. However, a negative correlation has been observed between angiogenic activity and mesh-induced inflammation in mice implanted with steroid-coated polyvinylidenfluoride (PVDF) meshes^29^. At menopause, decreased estrogen levels lead to a reduction in angiogenesis and therefore poor nourishment of vaginal tissue, which would increase the possibility of surgical complications.

#### Hormone replacement therapy

International guidance recommends conservative therapies as the first-line management of women with SUI, such as lifestyle changes and behavioral therapies, weight loss, and supervised pelvic floor muscle training^8,9^. Estrogens are believed to be beneficial in the treatment of SUI; starting estrogen replacement soon after menopause may be effective in preventing or delaying the onset of SUI^27,30^.

HRT is a common medical treatment used to supplement women with hormones that are lost during the menopausal transition. Conventional HRT includes an estrogen and progesterone component to relieve the menopausal syndrome^31^. In our study, patients with diagnosed menopausal syndrome who used HRT within a year prior to midurethral sling surgery had a significantly higher risk of surgical complications, especially urinary retention, at 5 years following midurethral sling surgery for SUI compared with their counterparts who did not have these risk factors. As patients diagnosed with menopausal syndrome commonly receive HRT to relieve their symptoms, it is difficult to distinguish between the effects of HRT and aging-related menopausal syndrome upon the risk of surgical complications associated with midurethral sling surgery. Although vaginal estrogen may be effective in preventing or delaying SUI, no evidence has shown that oral estrogen has benefits in SUI patients. A retrospective study in 2018 had found that the use of systemic estrogen may increase the SUI risk^32^. Estrogens are known to stimulate collagenase activity, which may lead to degradation of total collagen, especially the most supportive type I collagen, which may be replaced by weaker immature collagen^32^. The lack of mature collagen might lead to inadequate support of the vaginal and pelvic structure, especially postoperatively, increasing the possibility of recurrent SUI.

Estrogens may affect continence by several mechanisms, including increasing urethral resistance, raising sensory threshold of the bladder, increasing adrenoreceptor sensitivity in the urethral smooth muscle, and mediating relaxation of the detrusor muscle by adrenoceptor promotion^33,34^. Moreover, estrogen receptors are presented in the squamous epithelium of both proximal and distal urethra, and estrogen supplementary has been shown to improve the maturation index of urethral squamous epithelium^35^. Thus, the HRT application prior to the surgery may potentially exacerbate the continence and eventually cause urine retention.

#### AFR and voiding time

According to our univariable findings, AFR and voiding times from the urodynamic exam were worse in patients with surgical complications. Preoperative urodynamic evaluations may predict the risk of voiding dysfunction in women with SUI undergoing midurethral sling surgery^36^, although other research has failed to support such evaluations^37^. Another study from Taiwan has found that an abnormal preoperative uroflowmetry pattern and preoperative peak flow < 15 ml/s are risk factors contributing to voiding dysfunction following midurethral sling surgery^38^, and a low flow rate is reported by other researchers to be a risk factor for early voiding dysfunction postoperatively^39^. In our study, all of our patients (with and without surgical complications) had much lower AFR values (8.0 vs 10.0 ml/s, respectively) than the studies mentioned above. The discrepant outcomes may depend upon different criteria used by the various studies to determine low flow rates, as well as the differing severity of SUI in the various study populations.

### Study strengths and limitations

The strengths of this study include the careful efforts made to verify the study population to avoid selection bias. Because the sling type data were recorded only in unstructured text and could not be easily identified, an Obstetrics and Gynecology (OBGYN) physician (TFC) reviewed the operation records of all eligible patients to verify that these patients indeed received midurethral sling surgery. Another strength is that this study was able to examine the association between urodynamic parameters and surgical outcomes, as OBGYN physicians in CMUH regularly perform detailed urodynamic evaluations prior to surgery. Interestingly, a Cochrane review has concluded that urodynamic studies may change clinical decision making, but scant evidence shows that these evaluations improve clinical outcomes^40–42^. In our unadjusted analysis, we identified that lower average flow rate and longer voiding time were associated with surgical complications, which, if validated in another study, may serve as quantitative indicators for a high risk of surgical complications.

This study has several limitations. First, residual and unmeasured confounders could not be entirely excluded. For example, we may have misclassified patients with menopausal syndrome as being without menopausal syndrome, because we were reliant on ICD records from different doctors and from a retrospective single center database. Therefore, the effects of potential risk factors (such as patient lifestyle factors, physician bias and experience with recording menopausal symptoms) may have been slightly overestimated due to the underestimation of these positive confounders. Second, the standard protocol for follow-up is usually 6 months following surgery; we may have misclassified patients with surgical complications as instead belonging to the group without complications, because these patients may not come back to our hospital for care so were lost to follow-up. Third, the associations found in our study do not guarantee causality. Our findings were not verified in other populations under different healthcare systems and the results should be externally validated to prove generalizability.

## Conclusion

Our study found that a medication history of HRT was a risk factor for 5-year complications in women undergoing midurethral sling surgery for SUI. If this association is validated in future studies, it could provide guidance for surgeons, clinicians and patients assessing the risk of surgical complications when considering midurethral sling surgical treatment for SUI.

### Supplementary Information

Supplementary Information.
