# Supplementary material for: Risk factors for 5-year complications after midurethral sling surgery for stress urinary incontinence: a retrospective cohort study from Taiwan
Source: Sci Rep. 2023 Dec 5;13:21431. doi: 10.1038/s41598-023-48558-8 (PMC10697946; doi:10.1038/s41598-023-48558-8)
Supplement: Supplementary file 1 — Supplementary Information. [file 41598_2023_48558_MOESM1_ESM.docx]

**Supplementary Table 1. Definitions of comorbidities and diagnoses using the International Classification of Diseases (ICD) diagnosis codes and medications.**

|  | ICD-9-CM and ICD-10-CM |  | Medication |
| --- | --- | --- | --- |
| **Population definition** |  |  |  |
| Urinary incontinence | 625.6, 788.30, 788.33, 788.34, 788.39, N39.3, N39.41, N39.42, N39.46, N39.49, R32, R39.81 |  |  |
| **Comorbidity** |  |  |  |
| Diabetes mellitus | 250, E08-E13 | And | Insulin, Oral antidiabetic drugs (OADs) |
| Hypertension | 401-405, I10-I16 | And | Angiotensin-converting enzyme inhibitors (ACEIs), Angiotensin II receptor blockers (ARBs), Diuretics |
| Cardiovascular disease | 410-414, 430-438, 428, 429.2, 441, 442, G45-G46, I20-I25, I50, I60-I69, I71, I72, I77.7, I79.0 |  |  |
| Depression | 296.2, 296.3, 296.82, 298.0, 300.4, 301.12, 309.0, 309.1, 311.0, F06.31, F06.32, F25.1, F32, F33, F34.1, F43.21, F43.23 |  |  |
| Anxiety | 300, 308.3, 309.81, F06.4, F41.0, F41.1, F41.3, F41.8, F41.9 |  |  |
| Schizophrenia | 295, F20, F21, F25.9 |  |  |
| Affective psychosis | 296, 301.1, 311, F30-F33, F34.0, F34.1, F34.8, F34.9, F39, F60.89 |  |  |
| Organic psychosis | 290, 293, 294, F01-F06, F53 |  |  |
| Other psychosis | 295, 297-299, 301-302, 306-319, F07-F09, F20-F29, F34-F38, F40-F52, F54-F99 |  |  |
| Menopausal syndrome | 627.2, 627.4, 627.8, 627.9, N95.1, N95.8, N95.9 |  |  |
| Constipation | 564.0, K59.00, K59.01, K59.02, K59.09 |  |  |
| **Outcome definition** |  |  |  |
| Urine retention | 788.20, 788.29, R33.8, R33.9 |  |  |

**Supplementary Table 2. Hazard ratios (95% confidence intervals) of risk factors associated with 5-year surgical complications after midurethral sling surgery, combining menopause syndrome diagnosis or hormone replacement therapy as single variable.**

| **Characteristic** | **Available N** | **No. of events** | **HR (95% CI)** | |
| --- | --- | --- | --- | --- |
|  |  |  | **Crude model** | **Full model^a^** |
| Age (year) | 1961 | 93 | 1.034 (1.018, 1.050)* | 1.016 (0.992, 1.039) |
| Diabetes mellitus |  |  |  |  |
| No | 1783 | 80 | Ref | Ref |
| Yes | 178 | 13 | 1.642 (0.917, 2.942) | 1.552 (0.777, 3.099) |
| Surgical duration (h) | 1961 | 93 | 1.114 (0.985, 1.261) | 1.065 (0.894, 1.269) |
| Menopause syndrome diagnosis or HRT |  |  |  |  |
| No | 1541 | 55 | Ref | Ref |
| Yes | 420 | 38 | 2.629 (1.739, 3.976)* | 1.794 (1.033, 3.114)* |
| Average flow rate (ml/s) | 1846 | 90 | 0.904 (0.846, 0.965)* | 0.963 (0.890, 1.041) |
| TVT-O sling type |  |  |  |  |
| TOT | 620 | 19 | Ref | Ref |
| TVT-O | 1138 | 55 | 1.596 (0.949, 2.686) | 1.311 (0.726, 2.365) |

a. Full model was adjusted for age, diabetes mellitus, surgical duration, menopause syndrome diagnosis or hormone replacement therapy, average flow rate and TVT-O sling type (n=1653).

* Statistically significant.

*Abbreviations*: CI, confidence interval; HR, hazard ratio; HRT, hormone replacement therapy; TOT, transobturator tape; TVT-O, tension-free vaginal tape-obturator.

**Supplementary Table 3.** **Hazard ratios (95% confidence intervals) of risk factors associated with 5-year outcome of secondary surgery and with outcome of urinary retention after midurethral sling surgery.**

| **Characteristic** | **Available N** | **Secondary surgery** | | | **Urinary retention** | | |
| --- | --- | --- | --- | --- | --- | --- | --- |
|  |  | **No. of events** | **Crude HR (95% CI)** | **Full HR (95% CI)^a^** | **No. of events** | **Crude HR (95% CI)** | **Full HR (95% CI)^a^** |
| Age (year) | 1961 | 26 | 1.022 (0.991, 1.055) | 1.023 (0.974, 1.073) | 70 | 1.038 (1.020, 1.057)* | 1.013 (0.990, 1.037) |
| Diabetes mellitus |  |  |  |  |  |  |  |
| No | 1783 | 22 | Ref | Ref | 61 | Ref | Ref |
| Yes | 178 | 4 | 1.844 (0.633, 5.376) | 1.674 (0.572, 4.903) | 9 | 1.480 (0.738, 2.967) | 1.421 (0.590, 3.425) |
| Surgical duration (h) | 1961 | 26 | 0.888 (0.643, 1.226) | 1.070 (0.763, 1.501) | 70 | 1.166 (1.024, 1.328)* | 1.023 (0.833, 1.256) |
| Menopause syndrome diagnosis |  |  |  |  |  |  |  |
| No | 1767 | 23 | Ref | Ref | 57 | Ref | Ref |
| Yes | 194 | 3 | 1.188 (0.357, 3.951) | 1.430 (0.425, 4.81) | 13 | 2.102 (1.154, 3.829)* | 1.478 (0.684, 3.191) |
| Hormone replacement therapy |  |  |  |  |  |  |  |
| No | 1676 | 22 | Ref | Ref | 43 | Ref | Ref |
| Yes | 285 | 4 | 1.071 (0.369, 3.111) | 0.979 (0.237, 4.04) | 27 | 3.842 (2.376, 6.215)* | 1.904 (1.037, 3.496)* |
| Average flow rate (ml/s) | 1846 | 26 | 1.051 (0.974, 1.135) | 1.086 (0.974, 1.211) | 67 | 0.845 (0.781, 0.916)* | 0.899 (0.817, 0.989)* |
| TVT-O sling type |  |  |  |  |  |  |  |
| TOT | 620 | 11 | Ref | Ref | 8 | Ref | Ref |
| TVT-O | 1138 | 12 | 0.591 (0.261, 1.339) | 0.621 (0.232, 1.663) | 45 | 3.105 (1.465, 6.584)* | 2.389 (1.048, 5.448)* |

a. Full model: adjusted for age, diabetes mellitus, surgical duration, menopause syndrome diagnosis, hormone replacement therapy, average flow rate and TVT-O sling type (n=1653).

* Statistically significant.

*Abbreviations*: CI, confidence interval; HR, hazard ratio; HRT, hormone replacement therapy; TOT, transobturator tape; TVT-O, tension-free vaginal tape-obturator.
